# Awareness and Use of Folic Acid among Women of Childbearing Age

**DOI:** 10.5334/aogh.2396

**Published:** 2019-04-09

**Authors:** Giselle Medawar, Tarek Wehbe, Elizabeth Abou Jaoude

**Affiliations:** 1 The Holy Spirit University of Kaslik, Jounieh, LB; 2 The Lebanese Canadian and the Notre Dame University Hospitals, Department of Hematology, Jounieh, LB; 3 The Middle East Institute of Health Hospital, Department of Endocrinology, Bsalim, LB

## Abstract

Folic acid (FA) given before and during pregnancy reduces the risk of several birth defects, including neurologic, cardiac, urinary and other congenital anomalies in the newborn. Several studies around the world showed less than satisfactory awareness and intake of this important vitamin. We undertook the task of exploring this question among Lebanese women of childbearing age.

Between June and November 2014, we conducted this cross-sectional study on women aged 20 to 40 years. The subjects, who agreed to participate, completed a questionnaire, which included questions on sociodemographic characteristics, lifestyle, and knowledge about FA roles during pregnancy, as well as their babies’ health.

Seventy-six percent reported having knowledge about FA during pregnancy, but only a small proportion knew the benefits for taking it. Also, 93.9% of women took FA supplements during pregnancy, however only 33.6% of the participants took FA before becoming aware of their pregnancy.

Public health interventions to improve awareness of FA’s roles before and during pregnancy are crucial to curtail birth defects. It is our opinion that it is a very fruitful preventive medicine tool in which every community should invest.

## Introduction

FA, also known as vitamin B9, is an essential element for the formation of new blood cells, amino acids and DNA synthesis. The need for FA increases dramatically during pregnancy as new cells and DNA are forming. FA is found in many foods, especially leafy vegetables, wheat, eggs, cheese and some fruits like banana, kiwi, melon, apples and oranges. Biochemically, FA, or pteroyl-glutamic acid, consists of glutamate residues attached to a carboxyl terminus [1,2].

In 1992, the US Public Health Service Task Force recommended that all women who are able to become pregnant should take at least 400 micrograms (0.4 mg) of FA daily before conception and should continue this supplementation throughout the first trimester of pregnancy. For women who have had a previous pregnancy with neural tube defects (NTDs), FA supplementation of 4 mg daily should be taken during the same period [3,4,5,6].

NTDs are a group of debilitating and sometimes fatal conditions resulting from abnormal neural tube formation and closure. The most dramatic NTD is anencephaly, characterized by total or partial absence of the brain, and spina bifida or split spine resulting in mental and physical defects. NTDs are the second most common congenital group of defects in children after congenital heart defects. There is ample evidence that the presence of NTDs can be reduced if women receive FA supplementation in the preconception period [7,8]. NTDs, the risk of heart defects, urinary tract malformations and cleft lip and palate defects can all be significantly less prevalent when using FA supplementation during the neural tube closure; between the third and fourth week of gestation is crucial in this regard [9,10,11]. In addition to the birth defects, FA deficiency during pregnancy may increase the risk of preterm birth, low birth weight and intrauterine growth retardation of the fetus, and it may raise the level of homocysteine in the blood. High homocysteine levels may lead to spontaneous abortions, placental abruption and pre-eclampsia [12,13,14].

Despite this scientific body of evidence and the simple ways to reduce the incidence of all these defects, only a few women were found to be aware that FA should be started before pregnancy. Because the process of neural tube formation begins on the 19th day of conception, most women don’t become aware of their pregnancy early enough to start FA supplementation [13].

Starting FA supplementation before conception reduces the incidence of NTDs by 50–70%. FA supplementation may not prevent all neural tube defects, such as those related to chromosomal abnormalities and other mechanisms unrelated to FA deficiency, but it certainly have a major impact on its incidence [14,15]. Recent debates have focused on the need for mandatory fortification of cereal products, oral contraceptive pills or other common nutrients with FA. A number of countries continue to have a relatively high incidence of NTDs due to insufficient FA awareness and campaigns [1]. A high proportion of women worldwide, in both developed and developing countries, needs to be educated that FA should be taken before pregnancy and continued during the first three months after conception [13, 15,16].

One of those large awareness studies was run in 18 European countries on women aged 15–49. It screened 22,925 women and found 58% of them had at least one child and 38% of them had an unplanned pregnancy. Only 70% heard about FA, and 40% said they knew the benefits of this vitamin. Only 17% knew that FA can reduce the risk of neural tube deformities and that it needs to be taken before pregnancy [18].

Consulting a health care professional prior to pregnancy and complying with the required follow-up visits have been shown to help reduce the risks involved by 46% [17,18]. Even in developed countries, where women tend to make the necessary preconception visits and lifestyle changes, less than half of women were found to be taking FA before getting pregnant [9,19,20]. The long-term effectiveness of health promotion in the United States demonstrated that FA has a major role in the prevention of cleft lip and palate when taken in the first 12 weeks of pregnancy [12,21].

The objectives of this research are to evaluate the awareness among Lebanese women about FA and the association between FA supplementation with the risk of spontaneous premature birth and congenital malformations. We also looked at the question of awareness to start FA supplements before and during the conception period.

## Materials and Methods

We conducted this descriptive, cross-sectional, analytical study on women in the Kisrwan, Lebanon, area. Between June and November 2014, we approached five gynecologists randomly selected and requested to interview their patients aged 20–40 during their postnatal visits. Additionally, we took a group of 20 married women, outside the gynecologists’ offices, attempting to get pregnant as a control group.

A survey containing three sections was administered to the subjects: their sociodemographic characteristics, daily habits and pregnancy and child health. The sociodemographic issues included age during pregnancy, marital status, level of education and the economic situation. The lifestyle questions included vitamin and mineral supplements before or during pregnancy, knowledge about FA, their physical activities, health and eating habits, use of tobacco and alcohol before and during pregnancy, and medical monitoring of their health problems. Questions about pregnancy and child health included the planning for the pregnancy, birth weight and the baby’s health. The last question concerned the degree of trust and availability of various sources of information.

## Statistical Analysis

The Statistical Package for Social Sciences (SPSS) software version 16.0 was used. Frequencies, percentages, proportions, means and standard deviations were calculated. The values were considered significant at a p value less than 0.05. Descriptive statistics were used for the demographic and socioeconomic characteristics of the study population and to determine the proportion of women whose FA use is consistent with health professional guidelines. The comparison between women according to different demographic, socioeconomic characteristics and the history of pregnancy was done according to the type of each variable.

## Ethical Considerations

The protocol for this research was submitted to the Institutional Research and Ethics Board (IRB) of the Faculty of Agricultural and Food Sciences at the Holy Spirit University of Kaslik (USEK) and was initially approved on April 8, 2014. All subjects signed a written informed consent, and the identity of the subjects was kept confidential.

## Results

The questionnaires were completed between June and November 2014 by the group subjects. Three hundred ninety-three (393) women completed the questionnaires during their visits to the gynecologist (the group’s subjects), and twenty control subjects completed the survey outside the group (the control subjects). A total of 67.4% of group participants were aged between 26–35 years. The control subjects were 25–29 years old. Most of the group subjects (96.9%) were married, 80.9% were college educated and 83.2% had a job (Table 1). Only 33.6% of women took vitamins including FA before conception, whereas 93.9% took vitamins starting at the beginning of their pregnancy (Table 1). Of the participants who took FA before pregnancy, 55.3% took FA without any other supplement, the rest took it with other vitamins and only 85.7% took it on a daily basis.

**Table 1:** Basic demographics of the surveyed subjects.

		Total	Percentage (%)

Age at Pregnancy	20–25	86	21.9%
	26–30	160	40.7%
	31–35	105	26.7%
	36–40	42	10.7%
Marital Status	Single	0	0.0%
	Married	381	96.9%
	Widow	6	1.5%
	Divorced or separated	6	1.5%
Level of education	Elementary	20	5.1%
	High school	55	14.0%
	College level	318	80.9%
Economicstatus	Good	140	35.8%
	Moderate	233	59.6%
	Poor	18	4.6%
Work	Yes	327	83.2%
	No	66	16.8%
FA Intake before Pregnancy	Yes	132	33.6%
	No	261	66.4%
FA Intake during Pregnancy	Yes	369	93.9%
	No	24	6.1%
Did you take any vitamins including FA before getting pregnant?	FA alone	73	55.3%
	Multivitamin	19	14.4%
	Less than once weekly	2	1.6%
	1–3 times weekly	12	9.5%
	4–6 times weekly	4	3.2%
	Daily	108	85.7%
How often are you taking FA or vitamins with FA since the beginning of pregnancy?	FA alone	84	22.8%
	Multivitamin	220	59.6%
	Less than once weekly	0	0.0%
	1–3 times weekly	4	1.1%
	4–6 times weekly	19	5.2%
	Daily	344	93.7%

Of the group subjects, 76.59% reported hearing about FA, versus 68.3% in the control subjects. Of those who’d heard of FA 66.41% took it before pregnancy, and 93.89% took it during pregnancy. Most women who knew of FA did not know its benefits and how it would protect the child. Most of them answered, “It protects the health of the baby.” Some noted “It fixes the pregnancy and helps the development of the embryo” or “It is beneficial for the brain and the nerves.” Less than half of them answered “It prevents birth defects” or “It prevents neurologic problems.”

Among women who took FA before pregnancy, 51.5% never performed regular physical activity, 54.5% considered that their eating habits were good, 83.3% of the group subjects and 69.3% of the control subjects did not smoke, 63.6% did not drink any alcohol, 87.9% did not take medications and 90.9% denied any health problems.

During pregnancy, 53.7% of women who received FA did not engage in regular physical activity, and 52.8% reported lowering their alcohol and tobacco use. Also, 96.9% did not take other medications, and 93.9% did not have any health problems. The degree of knowledge correlated with the degree of education. Importantly, less-educated subjects were more likely to be smokers before and during pregnancy.

Only 26% of the group subjects (and no one in the control group) made a visit to a doctor while planning pregnancy. Of those who visited a gynecologist, only 54.5% were advised to take vitamins including FA in the preconception period but 72.9% noted that they took vitamins at the beginning of their pregnancy (Table 2).

**Table 2:** Preconception behavior of those taking FA supplements.

				Total	P(%)

Did you take vitamins in the months before getting pregnant?	Yes	Did you visit your physician before pregnancy to plan it?	Yes	72	54.5%
			No	60	45.5%
	No	Did you visit your physician before pregnancy to plan it?	Yes	30	11.5%
			No	231	88.5%
Did you take vitamins since you got pregnant?	Yes	Did you visit your physician before pregnancy to plan it?	Yes	100	27.1%
			No	269	72.9%
	No	Did you visit your physician before pregnancy to plan it?	Yes	2	8.3%
			No	22	91.7%

Among women who took vitamins and minerals during the preconception period, 77.8% had a child with a birth weight between three and four kilograms. None had a child with heart or other birth defects. However, among those who had not taken vitamins and minerals before the preconception period, 2.2% had a child with congenital malformations, including one case of spina bifida, one case of wrist agenesis and one case of musculoskeletal malformation.

As to the best source of information, 84.4% named their physician. Several other sources were named trustworthy, including the Internet (70.9%), friends (68.2%) and family (73.6%). Nearly half of the surveyed subjects noted that the dietitian was very helpful, and 59.3% chose the pharmacist. Only 10% considered radio, magazines and newspapers to be useful sources of information (Table 3).

**Table 3:** Optimal sources of information.

Source of Information	Utility	Percentage (%)

Doctors	313	84.4%
Dietetician	183	49.3%
Pharmacist	220	59.3%
Television	134	36.1%
Radio	40	10.8%
Revues	45	12.1%
Journals	42	11.3%
Internet	263	70.9%
Friends	253	68.2%
Familly	273	73.6%
Others	4	1.1%

## Discussion

In this study, awareness of the important health issues and necessary preparations before pregnancy among women leaves more to be desired. Both control and group subjects who knew of FA or received counseling from a health care professional before pregnancy were more likely to adopt positive behavioral changes before becoming pregnant, including FA intake and adopting a healthy diet. However, many more women did not get the needed FA and other health benefits. Most women consider quitting smoking and drinking alcohol three months before pregnancy as good habits to adopt, but the timing to start FA was not clear to them in order to reduce the risks to the fetus.

The prevalence of NTDs varies among countries. It ranges from 0.4 to 1.6 per 1,000 live births in Europe, 2.0 to 4.0 per 1,000 in the United States, 2.9 to 5.2 per 1,000 in the Middle East, and 4.0 to 9.0 per 1,000 live births in Turkey. The majority of these abnormalities could probably be prevented by the use of FA during the preconception period [22]. Our survey of 413 women showed that the majority were aware of the need for FA supplementation during pregnancy. Public health education about preconception supplementation remains an impactful issue to be addressed to protect against congenital defects. These results are consistent with many other studies conducted in Europe [16].

Studies out of the Middle East include a report from the Kingdom of Saudi Arabia, Taibah University, where the average knowledge score on FA usefulness among women who had previously been pregnant was 54.9%. A cross-sectional survey in Turkey found that 48.2% of the participants were aware that FA is necessary for the prevention of congenital anomalies. Even more significantly, although more than 88.2% of pregnancies were planned among women, only 14.2% of them reported using FA in the preconception period [1,23], Maher et al. showed that among a total of 603 participants visiting a gynecologist for the first time, about 25% of the cases had an unplanned pregnancy. Nearly 98% of women reported hearing about FA, but only 42% knew what it was preventing [24].

Among those who took alcohol and who smoked prior to pregnancy, a large proportion reduced alcohol and tobacco use in the preconception period. These results are consistent with a retrospective study in France, which showed that among 401 women who gave birth, 21.6% took an FA prescription, and 91.3% and 68.6% stopped using alcohol and tobacco, respectively. Surprisingly, only 13.8% of the 80.2% of women who had seen a doctor in the six months prior to conception discussed pregnancy planning during their visit, again emphasizing the lack of proper planning [25].

Similar to other studies, our survey showed that 48% of participants took FA supplements before pregnancy, 69% consumed alcohol and 71% had a preconception visit with a doctor. This study shows that a preconception visit was associated with an increase in FA supplementation, but this remains suboptimal. Over 70% of women reported planning their pregnancy, but only 33% had taken FA supplementation—a significant fact to consider in health awareness campaigns [19,20].

## Conclusion

This study underscores the need for better health education and greater involvement of health professionals in promoting essential information among women, especially in their preconception period. Promoting FA supplementation before pregnancy and during the childbearing ages is essential for any community. This issue takes a particularly heightened importance among the moderately educated, low-income women, as well as those with risk factors such as obesity, epilepsy, diabetes, history of NTDs and with a risky lifestyle (including smoking and alcohol consumption).

Many women in this survey were unaware of the importance of taking FA during the preconception period to reduce several preventable congenital risks. Like most awareness-raising campaigns, each community should devise its own methods and approaches to this problem, which remains significant even in developed countries. Promoting preconception consultations is one necessary objective to consider in any public health campaign targeting women use of FA in their childbearing ages [26].

Our data in Lebanon implies that physicians, pharmacists and dieticians can have a leading role in disseminating the information about the need for FA and the behavioral modifications as pregnancies are being planned or occurring “accidentally.” The recommended dose of 400 μg of FA before conception and during the first three months of pregnancy should be widely distributed. This is one more example of prevention, education and awareness capable of saving huge societal and economic burdens.

## Additional File

The additional file for this article can be found as follows:

10.5334/aogh.2396.s1 Awareness and Use of Folic Acid among Women of Childbearing Age. Additional Raw Data.

## Figures and Tables

**Table:** 

FA	folic acid
T2D	type 2 diabetes
NTD	neural tube defect
