# Supplementary material for: Awareness and Use of Folic Acid among Women of Childbearing Age
Source: Ann Glob Health. 2019 Apr 9;85(1):54. doi: 10.5334/aogh.2396 (PMC6634342; doi:10.5334/aogh.2396)
Supplement: Awareness and Use of Folic Acid among Women of Childbearing Age. — Additional Raw Data. [file agh-85-1-2396-s1.pdf]

Awareness of the Importance of Folic Acid Among Lebanese Women

AUTHORS: Giselle Medawar [1], Tarek W. Wehbe [2], Elizabeth Abou Jaoude M.D.[3]

Results

quête s'est déroulée entre les mois de Juinet Novembre 2014 par le biais d'un tionnaire donné aux patientes lors de leurs visites postnatales. Un total de 393 femmes nt interrogées, lors de leur visite aux gynécologues, afin de répondre au questionnaire.

au 2. Caractéristiques socio-démographiques des participantes.

		otal	P (%)
âge aviez-vous lors de votre grossesse?	5	86	21.9%
	0	160	40.7%
	5	105	26.7%
	0	42	10.7%
est votre statut?	bataire	0	.0%
	ée	381	96.9%
	ve	6	1.5%
	rcée/ Séparée	6	1.5%
est votre niveau d'éducation?	es primaires	20	5.1%
	es secondaires	55	14.0%
	es universitaires	318	80.9%

ıbleau 2 décrit les caractéristiques socio-démographiques des participantes. 67.4% des femmes ont ꜗ 26 et 35 ans, 96.9% sont mariées, 80.9% ont un niveaud'études universitaires et83.2% travaillent. retrospective study of Keserwan provides an overview of the period of supplementation: 33.6% of ıen took vitamins and minerals before conception and 93.9% took vitamins and minerals since the nning of their pregnancy (Table 3). Table 4 shows that 55.3% of women took folic acid alone as lementation during the pre-pregnancy period, 85.7% of whom took it daily. While 59.6% of cipants took multivitamins containing folic acid as supplementation and a large proportion took them ꜗ (93.7%). **Tableau 4.** Prise d'acide folique seul ou sous-forme de multivitamines et sa fréquence

				Total	P (%)
ɛ-vous pris des ıines et des minéraux ıt le mois précédant la ɛesse?		eFolique	e Folique Seul	73	55.3%
		ivitamine	ivitamine	19	14.4%
		elle fréquence les ɛez-vous?	ıs d'une fois par semaine	2	1.6%
			fois par semaine	12	9.5%
			fois par semaine	4	3.2%
			les jours	108	85.7%
ıis le début de votre ɛesse, avez-vous ınué ou commencé à dre des vitamines etdes raux?		eFolique	e Folique Seul	84	22.8%
		ivitamine	ivitamine	220	59.6%
		elle fréquence les ɛez-vous?	ıs d'une fois par semaine	0	.0%
			fois par semaine	4	1.1%
			fois par semaine	19	5.2%
			les jours	344	93.7%

1.1.3 Les connaissances sur l'acide folique

More than half of the participating women heard about folic acid (76.5%) and among those who did not hear this vitamin, 8.7% knew it should be taken before and during pregnancy (Table 5).).

Table 5. Knowledge about folic acid

Total P (%)

Have you ever heard of folic acid? Yes According to you, when is it most beneficial in a woman's life to take folic acid? Before and during pregnancy 230 76.4%

Before pregnancy 6 2.0%

During pregnancy 59 19.6%

Do not know 6 2.0%

No How do you think it's most beneficial in a woman's life to take folic acid? Before and during pregnancy 8 8.7%, During pregnancy 6 6.5%, Do not know 78 84.8%

Figure 3 shows that 76.59% of the participants have knowledge about folic acid, 66.41% have been supplemented with this vitamin before pregnancy and 93.89% have taken this vitamin during pregnancy. In fact, among the women who have heard of folic acid, a very low proportion knew its benefits and in what medical conditions this vitamin would protect the child. Most of them answered: "It protects the health of the baby", some noted: "It fixes the pregnancy and helps the development of the embryo", "It is beneficial for the brain and the nerves" and less half of them answered: "It prevents birth defects", "It prevents Spina Bifida from the infant".

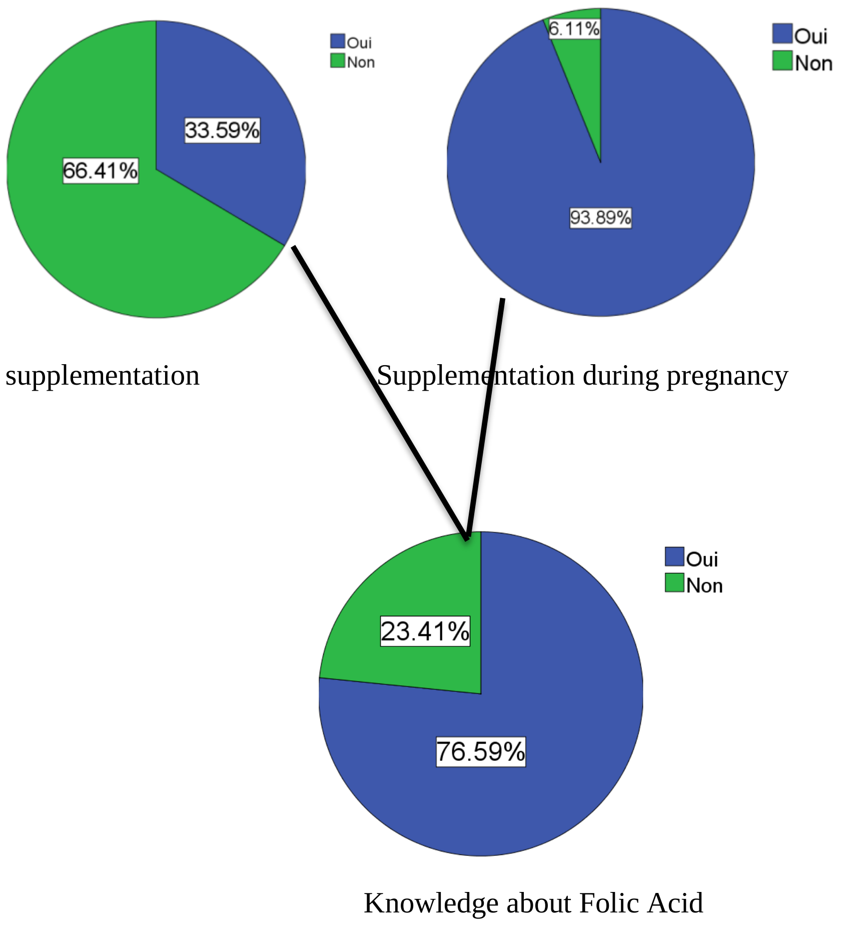

Figure 3. Les proportions des femmes selon leurs connaissances sur l'acide folique, leur supplémentation en période préconceptionnelle et leur supplémentation durant la grossesse

Table 6 shows that among women who took vitamins and minerals before pregnancy, 51.5% had never had any physical activity, 54.5% considered that their eating habits were good, 83.3% did not smoke, 63.6% did not smoke. drank no alcohol, 87.9% did not take medication and 90.9% did not have any health problems.

During pregnancy, 53.7% of women who were supplemented did not engage in physical activity, 52.8% had good eating habits, and a large proportion decreased their use of alcohol and tobacco.

Table 6. Lifestyles of participants supplemented during the preconception period

z-vous pris des mines et des éraux durant le s précédant la ssesse?				Total	P (%)
		nbien de fois avez-vous iqué des activités physiques 20 à 30 minutes par séance, s vos temps libres, au cours 3 derniers mois avant votre ssesse?	une fois	68	51.5%
			iron 1 fois/mois	16	12.1%
			iron 2 à 3 fois par s	18	13.6%
			iron 1 fois par aine	8	6.1%
			iron 2 fois par aine	4	3.0%
			iron 3 fois par aine	10	7.6%
			fois ou plus par aine	8	6.1%
		nparativement à d’autres sonnes de votre âge, diriez-s que vos habitudes nentaires sont en général:	ellentes	18	13.6%
			nes	72	54.5%
			yennes	32	24.2%
			uvaises	10	7.6%
		ant les 12 mois précédant e grossesse fumiez-vous?	, régulièrement	2	1.5%
			, occasionnellement	20	15.2%
			n	110	83.3%
		ool		48	36.4%
			n	84	63.6%
		nt votre grossesse, preniez-s des médicaments?		16	12.1%
			n	116	87.9%
		nt votre grossesse, étiez-s suivie régulièrement par e médecin pour un problème anté?		12	9.1%
			n	120	90.9%

Among those who did not take pre-conceptional supplementation, almost half did not engage in physical activity, had good eating habits, and decreased alcohol and tobacco use. 96.9% did not take medication and 93.9% did not have a health problem (Table 7).

eau 7. Les habitudes de vie des participantes sans supplémentation avant la période onceptionnelle

				Total	P (%)
-vous pris itamines et raux durant le précédant la esse?		bien de fois avez-vous qué des activités physiques 0 à 30 minutes par séance, vos temps libres, au cours 3 derniers mois avant votre esse?	ıne fois	148	56.7%
			ron 1 fois/mois	16	6.1%
			ron 2 à 3 fois par mois	26	10.0%
			ron 1 fois par semaine	6	2.3%
			ron 2 fois par semaine	16	6.1%
			ron 3 fois par semaine	29	11.1%
			s ou plus par semaine	20	7.7%
		parativement à d'autres onnes de votre âge, diriez- que vos habitudes entaires sont en général:	llente	32	12.3%
			ne	139	53.3%
			enne	66	25.3%
			vaise	24	9.2%
		d'alcool	lcool	131	50.2%
			ol	130	49.8%
		nt les 12 mois précédant grossesse fumiez-vous?	régulièrement	14	5.4%
			occasionnellement	39	14.9%
				208	79.7%
		ıt votre grossesse, preniez- des médicaments?		8	3.1%
				253	96.9%
		ıt votre grossesse, étiez- suivie régulièrement par médecin pour un problème nté?		16	6.1%
				245	93.9%

More than half of the women who did not take supplementation during pregnancy, did not engage in physical activity (75.0%), had good eating habits, and decreased their use of tobacco and alcohol.

According to our survey, it was found that 74% of women did not consult a physician before their pregnancy for planning and among those who planned to get pregnant, 61.2% consulted their doctor before 1 to 6 months (Table 8).

eau 8. La planification de la grossesse

		Total	P (%)
-vous consulté un médecin avant votre esse dans le but de planifier celle-ci?	Oui	102	26.0%
	Non	291	74.0%
	**Total**	393	100.0%
	0-6	60	61.2%
	7-12	17	17.3%
	13-24	14	14.3%
	25-36	7	7.1%
	**Total**	98	100.0%

In fact, Table 9 shows that 54.5% of women who had planned their pregnancy were advised to take vitamins and minerals in the preconception period and 11.5% of those who had planned to get pregnant did not take vitamins nor of minerals before their pregnancy. In addition, women who had not planned (72.9%) noted that they had been taking vitamins and minerals since the beginning of their pregnancy.

eau 9. Le comportement avant la grossesse des participantes avec supplémentation

				Total	P(%)
-vous pris des vitamines s minéraux durant le mois édant la grossesse?		-vous consulté un ecin avant votre grossesse le but de planifier celle-		72	54.5%
				60	45.5%
		-vous consulté un ecin avant votre grossesse le but de planifier celle-		30	11.5%
				231	88.5%

is le début de votre esse, avez-vous continué mmencé à prendre des nines et des minéraux?		-vous consulté un ecin avant votre grossesse le but de planifier celle-		100	27.1%
				269	72.9%
		-vous consulté un ecin avant votre grossesse le but de planifier celle-		2	8.3%
				22	91.7%

3.1.1.6 Malformations in the newborn

Among women who took vitamins and minerals during the preconception period, 77.8% had a child with a birth weight between 3 and 4 kg. None had a child with heart problems or with birth defects. But, among those who had not taken vitamins and minerals before the preconception period, 60% had a child weighing 3 to 4 kg. None had a child with heart problems and 2.2% had a child with congenital malformations including one case of Spina Bifida, cases of wrist agenesis, cases of musculoskeletal malformations, and one case of jaundice and jaundice. (Table 10)

leau 10. Les caractéristiques du nouveau-né lors de grossesse planifiée et supplémentée

				Total	P(%)
-vous pris des nines et des minéraux nt le mois précédant la esse?		s		2	2.8%
				14	19.4%
				56	77.8%
		e enfant a-t-il des lèmes cardiaques?		0	.0%
				132	100.0%
		e enfant présente-t-il des ormations congénitales?		0	.0%
				132	100.0%
		s		6	20.0%
				6	20.0%
				18	60.0%
		e enfant a-t-il des lèmes cardiaques?		0	0%
				261	100%
		e enfant présente -t-il des		8	3.1%

		ormations congénitales?		253	96.9%
is le début de votre esse, avez-vous nué ou commencé à dre des vitamines et raux?		s	)	6	6.0%
			)	20	20.0%
				74	74.0%
		e enfant a-t-il des lèmes cardiaques?		0	0%
				369	100%
		e enfant présente -t-il des ormations congénitales?		8	2.2%
				361	97.8%
		s	)	2	100.0%
			)	0	.0%
				0	.0%
		e enfant a-t-il des lèmes cardiaques?		0	0%
				24	100%
		e enfant présente -t-il des ormations congénitales?		0	.0%
				24	100.0%

3.1.1.7The usefulness of promoting supplementation by information sources

According to the participants, the largest proportion (84.4%) considered that the doctor is a very useful source of information for the promotion of health information, a large proportion gave usefulness to the internet, to friends, to family, 70.9%, 68.2%, 73.6%, respectively. Nearly half noted that the dietitian was very helpful, 59.3% found the pharmacist more useful, almost only 10% consider that radio, magazines and newspapers are very useful. (Table 11).

leau 11. Utilité des sources d'information de santé chez les participantes

	Utilité	P (%)
ecin	313	84.4%
éticienne	183	49.3%
macien	220	59.3%
vision	134	36.1%
o	40	10.8%

[illegible]ıes	45	12.1%
[illegible]ıaux	42	11.3%
[illegible]net	263	70.9%
[illegible]s	253	68.2%
[illegible]ille	273	73.6%
[illegible]es	4	1.1%
**Total**	**1770**	**477.1%**

3.1.2Gynecologists' questionnaire

The gynecologists we met consider that supplementation with folic acid before pregnancy is beneficial. They consider that this supplementation should begin 3 months before pregnancy and should be given at a dose of 400 micrograms per day (Table 12).

Table 12. Gynecologists' views on supplementation and folic acid intake before pregnancy

		**Total**	**P(%)**
[illegible]tre avis, la supplémentation en acide [illegible]ue avant la grossesse est-elle [illegible]fique ?		5	100.0%
		0	0%
[illegible]bien de mois		5	100.0%
[illegible]ıence		5	100.0%

Similarly, the gynecologists in this survey recommend folic acid supplementation before and during pregnancy. All gynecologists included in this study are confident that preceptive folic acid supplementation decreases congenital malformations in the newborn.

In addition, these gynecologists inform their pregnant patients about the risks and benefits of folic acid supplementation (80% through prenatal consultation and 20% through brochures at the clinic) (Table 13).

[illegible]eau 13. Les sources d’information pour les femmes chez les gynécologues

		Total	P (%)
[illegible]mez-vous vos patientes		5	100.0%

ntes sur les risques et les tages de la lémentation en acide ue avant et durant la sesse ?		0	.0%
vers	brochures à la clinique	1	20.0%
	medias sociaux	0	.0%
	sultation prénatale	4	80.0%
